# Honokiol protects rat hearts against myocardial ischemia reperfusion injury by reducing oxidative stress and inflammation

**DOI:** 10.3892/etm.2012.766

**Published:** 2012-10-25

**Authors:** YUN WANG, ZHONG-ZE ZHANG, YUN WU, JIA ZHAN, XIANG-HU HE, YAN-LIN WANG

**Affiliations:** Department of Anesthesiology, Zhongnan Hospital of Wuhan University, Wuhan, Hubei 430071, P.R. China

**Keywords:** honokiol, ischemia/reperfusion, oxidative stress, inflammation, NF-κB

## Abstract

Honokiol, a potent radical scavenger, has been demonstrated to ameliorate cerebral infarction following ischemia/reperfusion (I/R) injury. However, its effects on myocardial I/R injury remain unclear. The present study aimed to examine the effects of honokiol on myocardial I/R injury and to investigate its potential cardioprotective mechanisms. Sprague-Dawley rats were pretreated with honokiol and exposed to a 30-min myocardial ischemia followed by 2-h coronary reperfusion. Myocardial I/R-induced infarct size and biochemical and histological changes were compared. The expression of nuclear factor κB(NF-κB; p65) was assessed by western blotting. Pretreatment with honokiol significantly reduced infarct size, and serum creatine kinase (CK) and lactate dehydrogenase (LDH) release compared with those in the I/R group following a 2-h reperfusion. The malondialdehyde (MDA) level, myeloperoxidase (MPO) activity, concentrations of tumor necrosis factor (TNF)-α and interleukin (IL)-6 and expression level of NF-κB were all reduced by honokiol pretreatment, while honokiol inhibited the decreases in superoxide dismutase (SOD) and catalase (CAT) activities. In addition, less neutrophil infiltration and histopathological damage in the myocardium were observed in the honokiol-pretreated group. These findings indicate that honokiol pretreatment diminished myocardial I/R injury through attenuation of oxidative stress and inflammation.

## Introduction

Myocardial infarction following ischemia is a major cause of mortality worldwide. Although early reperfusion is necessary for myocardial salvage, reperfusion itself exacerbates myocardial injury (1,2). Myocardial ischemia reperfusion (I/R) injury causes negative events such as local inflammation and free radical-mediated lipid peroxidation (3,4). The latter is associated with deleterious effects on reversible injuries of myocardial cells (5) and contributes to cardiac dysfunction (6).

It is known that an increased formation of reactive oxygen species (ROS) at the time of reperfusion plays a crucial role in driving the inflammatory cascade (7). ROS activate nuclear factor κB (NF-κB), a key transcription factor, and modulate the expression of inflammation-related proteins, including tumor necrosis factor (TNF)-α and interleukin (IL)-6, which are significant in the development of myocardial I/R injury (8). With this background, pharmacological intervention to scavenge ROS may have a double benefit in myocardial I/R injury: it may diminish the progression of myocardial damage by either attenuating ROS-induced lipid peroxidation or by preventing the free radical-mediated inflammation response.

Honokiol is an active component of *Magnolia officinalis* (Houpo), a traditional Chinese herb used for the treatment of various vascular diseases, including ischemia, heart disease and stroke (9). Honokiol has been shown to mitigate cerebral I/R injury through its antioxidant and anti-inflammatory effects (10,11). Early studies have also shown that honokiol is able to limit infarct size and display anti-arrhythymic effects in rats with acute myocardial infarction (12,13). However, the mechanism remains unclear and there have been few studies concerning honokiol in myocardial I/R injury. The present study was designed to examine the effects of honokiol in a rat model of myocardial I/R injury *in vivo*, and to investigate its potential cardioprotective mechanisms.

## Materials and methods

### Animals

Sprague-Dawley rats (male, 250±20 g) were purchased from Wuhan University Animal Center (Wuhan, China). This study conformed to the Guide for the Care and Use of Laboratory Animals of the National Institutes of Health (NIH Publication No. 80-23), and the experimental procedures were approved by the Institutional Animal Ethics Committee of Wuhan University.

### In vivo myocardial I/R model

The *in vivo* myocardial I/R model was modified from a previous study (14). Briefly, rats were anesthetized with 1% pentobarbital sodium (40 mg/kg, intraperitoneally). The rats were intubated and mechanically ventilated with room air using a rodent respirator (DV-2000, Shanghai Jia Peng Technology Co., Ltd., Shanghai, China). A left thoracotomy was carried out to expose the heart. A 5-0 silk ligature was then passed under the left anterior descending (LAD) coronary artery, and a small vinyl tube was placed on top of the vessel to form a snare for the reversible coronary occlusion. After a 30-min ischemia, the heart was reperfused for 2 h by releasing the snare.

### Experimental groups and protocols

A total of 50 rats were randomly divided into four groups: sham (n=10), sham plus honokiol (sham-HK; n=10), I/R (n=15) and I/R plus honokiol (I/R-HK; n=15). Honokiol (5 mg/kg; Sigma-Aldrich, Inc., St. Louis, MO, USA) dissolved in dimethyl sulfoxide (DMSO) was intraperitoneally administered to the rats 30 min prior to ischemia. The dose of honokiol was administered as described previously (15,16).

### Determination of infarct size

At the end of the experiment, the ligature around the LAD was retied. A 2-ml dose of 2% Evans blue dye (Sigma-Aldrich, Inc.) was administered intravenously to distinguish between the perfused and non-perfused regions. The heart was separated rapidly and cut into 3-mm thick slices parallel to the atrioventricular groove. The slices were then placed in 1% tetrazolium chloride solution (TTC; Sigma-Aldrich, Inc.) for 20 min to identify the infarct zone. The weight of the infarct area (white) and the area at risk (AAR; red) were measured. The infarct size was expressed as a percentage of AAR mass.

### Measurement of serum levels of creatine kinase (CK) and lactate dehydrogenase (LDH)

Following a 2-h reperfusion, blood samples were obtained and centrifuged. The serum levels of CK and LDH were measured by a colorimetric method, using commercial kits (Nanjing Jiancheng Bioengineering Institute, Nanjing, China) according to the manufacturer’s instructions. The recorded values are presented in U/l.

### Assay of malondialdehyde (MDA), superoxide dismutase (SOD), catalase (CAT) and myeloperoxidase (MPO)

The frozen samples of the ischemic zone were homogenized and centrifuged. Subsequently, the supernatants were collected for analysis of the MDA level, and SOD, CAT and MPO activities. The measurements were obtained spectrophotometrically with commercially available kits (Nanjing Jiancheng Bioengineering Institute) according to the manufacturer’s instructions. The MDA level was represented in nmol/mg protein. The activities of SOD and CAT were expressed as U/mg protein. MPO activity was expressed as U/mg wet tissue.

### Determination of TNF-α and IL-6

The TNF-α and IL-6 levels in the cardiac tissues were measured by enzyme-linked immunosorbent assay using specific kits (Boster Biological Technology Company, Wuhan, China) according to the manufacturer’s instructions. Values are expressed as pg/mg protein.

### NF-κB (p65) expression

The expresssion of NF-κB (p65) was evaluated by western blotting. Ischemic cardiac tissues were homogenized and nuclear proteins were extracted with a Nuclear-Cytosol Extraction kit (Applygen Technology Inc., Beijing, China) according to the manufacturer’s instructions. Once the protein concentration had been determined by a bicinchoninic acid protein assay (Beyotime Biotechnology, Inc., Nantong, China), proteins were separated on 15% SDS-polyacrylamide gels, transferred to a nitrocellulose membrane, probed with the primary antibodies against NF-κB (p65; 1:500; Santa Cruz Biotechnology, Inc., Santa Cruz, CA, USA) and β-actin (1:500; Santa Cruz Biotechnology, Inc.) and then incubated with a goat anti-rabbit secondary antibody (1:1,000; Santa Cruz Biotechnology, Inc.). Proteins bands were then visualized with an enhanced chemiluminescence kit (Beyotime Biotechnology, Inc.).

### Histopathological examination

Cardiac tissues were fixed in 10% formalin and embedded in paraffin. The embedded tissues were sectioned and stained with hematoxylin and eosin (H&E) for observation of any myocardial neutrophil infiltration.

### Statistical analysis

All results were expressed as the mean ± SD. Differences between groups were analyzed by one-way ANOVA followed by Bonferroni’s post hoc test. P<0.05 was considered to indicate a statistically significant difference.

## Results

### Myocardial infarct size

**Figure 1:**
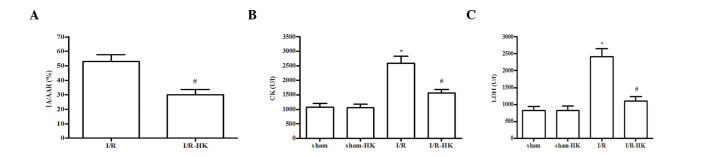
Honokiol attenuates I/R-induced myocardial necrosis. (A) Infarct size was expressed as a percentage of AAR mass (n=5). (B) Effect of honokiol on serum levels of CK (n=8). (C) Effect of honokiol on serum levels of LDH (n=8). ^*^P<0.05 vs. the sham group, ^#^P<0.05 vs. the I/R group. HK, honokiol; I/R, ischemia/reperfusion; IS, infarct size; AAR, area at risk; CK, creatine kinase; LDH, lactate dehydrogenase.

Following a 2-h reperfusion, honokiol treatment significantly reduced myocardial infarct size compared with that in the I/R group (P<0.05; Fig.1A). No significant difference in AAR was observed between the two groups (data not shown).

### Serum levels of CK and LDH

The serum levels of CK and LDH were markedly elevated in the I/R group compared with those in the sham group (P<0.05). However, the elevation in CK and LDH levels was significantly suppressed by honokiol adminstration (I/R-HK vs. I/R, P<0.05). Honokiol did not affect the serum levels of LDH and CK in the sham group (sham-HK vs. sham, P>0.05; Fig. 1B and C).

### MDA, SOD, CAT and MPO in cardiac tissues

The MDA level and MPO activity were notably increased, while SOD and CAT activities were notably decreased in cardiac tissues exposed to I/R injury (P<0.05). Honokiol attenuated the I/R-induced increases in the MDA level and MPO activity, while it inhibited the decreases in SOD and CAT activities. Honokiol did not affect the MDA, SOD, CAT and MPO levels in the sham group (sham-HK vs. sham, P>0.05; Table I).

### Levels of TNF-α and IL-6 in the myocardium

**Figure 2:**
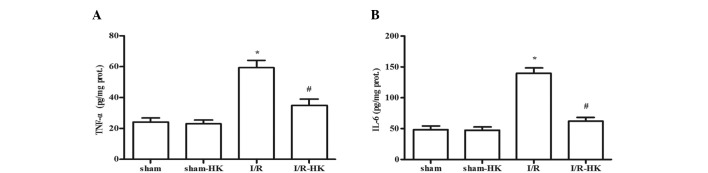
Effect of honokiol on the levels of (A) TNF-α and (B) IL-6 in cardiac tissues following I/R (n=5). ^*^P<0.05 vs. the sham group, ^#^P<0.05 vs. the I/R group. HK, honokiol; TNF-α, tumor necrosis factor-α; IL-6, interleukin-6; I/R, ischemia/reperfusion.

TNF-α and IL-6 levels were greatly induced by I/R compared with those in the sham group (P<0.05). Honokiol significantly suppressed the increases in TNF-α and IL-6 (P<0.05) levels. The TNF-α and IL-6 levels in the sham group were not altered by the honokiol treatment (sham-HK vs. sham, P>0.05; Fig. 2).

### NF-κB (p65) expression

**Figure 3:**
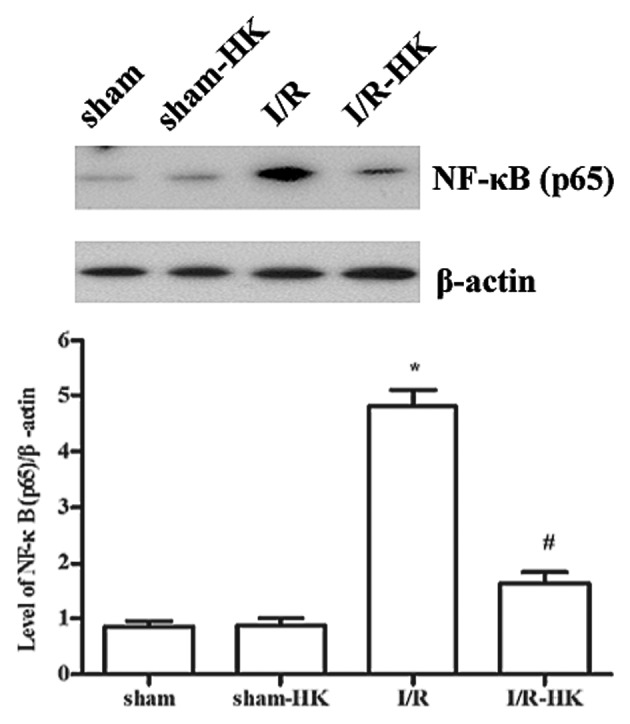
Effect of honokiol on NF-κB (p65) expression levels (n=5). ^*^P<0.05 vs. the sham group, ^#^P<0.05 vs. the I/R group. HK, honokiol; NF-κB, nuclear factor κB; I/R, ischemia/reperfusion.

NF-κB expression was significantly upregulated following I/R injury, however treatment with honokiol significantly decreased the NF-κB expression (P<0.05). Honokiol did not affect the expression of NF-κB in the sham group (sham-HK vs. sham, P>0.05; Fig. 3).

### Histopathological examination of myocardial tissues

**Figure 4:**
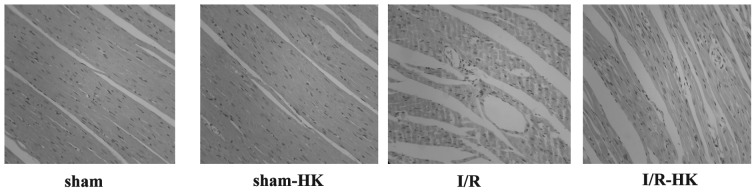
Histopathological changes in rat cardiac tissue (hematoxylin and eosin, x400). The sham and sham-HK groups showed normal tissue structure; the I/R group showed widespread myocardial structure disorder, perivascular edema and neutrophil infiltration; I/R-HK showed mild structural damage and interstitial edema. HK, honokiol; I/R, ischemia/reperfusion.

No histological lesions were observed in the sham or sham-HK groups. In the I/R group, there was evident structural disorder, perivascular edema and neutrophil infiltration. However, honokiol treatment markedly suppressed the I/R-induced myocardial injury and neutrophil infiltration (Fig. 4).

## Discussion

The major findings of the present study are as follows. Firstly, honokiol pretreatment played a protective role against myocardial I/R injury. Secondly, the protective effects of honokiol during myocardial I/R injury were correlated with the attenuation of oxidative stress and inflammation. Thirdly, the antioxidant and anti-inflammatory effects of honokiol in myocardial I/R may be due to the inhibition of the NF-κB pathway.

As a component of a traditional Chinese herb used in various vascular diseases, honokiol has been demonstrated to protect heart mitochondria against lipid peroxidation and to diminish the myocardial infarct size of rats exposed to coronary occlusion (12,17). However, whether honokiol has beneficial effects on myocardial I/R remains unclear. The present study revealed that honokiol pretreatment was able to protect rats against myocardial I/R injury, as evidenced by a smaller infarct size and reduced amounts of CK and LDH being released.

It is well documented that ROS play a pivotal role in the development of myocardial I/R injury. ROS cause direct damage to proteins and membranes as well as indirect damage through activation of pro-apoptotic pathways (3). SOD and CAT, being key free radical scavenging enzymes, are the primary line of defense against tissue damage induced by ROS (18). MDA, an significant product of lipid oxidation, is often used to evaluate free radical-mediated myocardial cell injury (19). Reducing the elevation of MDA level and suppressing the decrease of SOD and CAT activities has cardioprotective effects in the ischemic-reperfused myocardium (20). The present study revealed that honokiol adminstration significantly reduced the elevation of MDA level and attenuated the decrease of SOD and CAT activities in ischemic-reperfused hearts. These data suggest that honokiol may attenuate myocardial I/R-induced oxidative stress, which thus contributes to the attenuation of myocardial damage.

Studies have demonstrated that inflammation is associated with myocardial reperfusion injury, which is triggered by ROS accumulation (21). Pro-inflammatory cytokines and neutrophils play important roles in the cardiac inflammatory response to I/R (4). TNF-α and IL-6 are pro-inflammatory cytokines which contribute significantly to myocardial dysfunction (22). Neutrophils are able to release oxidants and proteases that damage tissues (23). Neutrophils also induce inflammatory mediators that amplify neutrophil recruitment in the ischemic-reperfused myocardium, thus expanding myocardial damage (23). MPO is a neutrophil protein whose activity may be considered to be an indicator for neutrophil infiltration in injured tissues (24). In the present study, the generation of TNF-α and IL-6, neutrophil infiltration and MPO activity were all increased significantly in the rat hearts following I/R injury. Each of these changes were inhibited by honokiol pretreatment. This suggests that honokiol protected the myocardium from inflammation during I/R insult. Such protection may contribute to the survival of the myocardial cells, as demonstrated by our results.

NF-κB (p65) is an early redox-sensitive transcriptional factor which regulates the inflammation-related gene expression involved in myocardial I/R (25). NF-κB activation induces pro-inflammatory proteins and neutrophil infiltration, resulting in injury to endothelial and myocardial cells (26). Blocking NF-κB activation reduces myocardial infarcts following I/R injury (27,28). Honokiol has been reported to exert anti-inflammatory effects and protect endothelial cells through the inhibition of the NF-κB pathway (29,30). In the present study, pretreatment with honokiol notably reduced myocardial I/R-induced NF-κB activation, which was associated with the attenuation of oxidative stress and inflammation. These results indicate that the suppression of NF-κB by honokiol may be a potential mechanism of its cardioprotective action.

In summary, the present study showed that honokiol had a protective effect in a rat model of myocardial I/R injury, possibly by reducing oxidative stress and inflammation. However, only the beneficial role during the early phase of myocardial reperfusion was investigated. Future studies to investigate the effects of honokiol on heart failure and long-term survival postinfarction would be worthwhile.

## Figures and Tables

**Table I:** Effects of honokiol on MDA level and SOD, CAT and MPO activities in cardiac tissues following I/R.

Group	MDA (nmol/mg protein)	SOD (U/mg protein)	CAT (U/mg protein)	MPO (U/mg wet tissue)
Sham	2.3±0.4	154.2±9.6	55.4±4.1	1.3±0.2
Sham-HK	2.4±0.2	156.8±8.6	58.3±3.2	1.2±0.2
I/R	5.9±0.7^a^	80.3±7.9^a^	25.2±4.0^a^	2.9±0.3^a^
I/R-HK	3.3±0.4^b^	137.1±12.0^b^	44.6±4.8^b^	1.8±0.2^b^

Data are expressed as mean ± SD, n=5 in each group. HK-treated groups were injected intraperitoneally with HK. At 2 h after I/R, the MDA level and SOD, CAT and MPO activities in cardiac tissues were measured.

a P<0.05 vs. the sham group,

b P<0.05 vs. the I/R group. HK, honokiol; MDA, malondialdehyde; SOD, superoxide dismutase, CAT, catalase; MPO, myeloperoxidase, I/R, ischemia/reperfusion.
